# Expression and Secretion of an Atrial Natriuretic Peptide in Beige-Like 3T3-L1 Adipocytes

**DOI:** 10.3390/ijms20246128

**Published:** 2019-12-05

**Authors:** In-Seon Bae, Sang Hoon Kim

**Affiliations:** Department of Biology, College of Sciences, Kyung Hee University, Seoul 02447, Korea; since89_bis@naver.com

**Keywords:** atrial natriuretic peptide, beige adipocytes, docosahexaenoic acids

## Abstract

The browning of white adipose tissue (beige adipocytes) stimulates energy expenditure. Omega-3 fatty acids have been shown to induce thermogenic action in adipocytes via G-protein coupled receptor 120 (GPR120). Atrial natriuretic peptide (ANP) is a peptide hormone that plays the role of maintaining normal blood pressure in kidneys to inhibit Na^+^ reuptake. Recently, ANP was found to induce adipocyte browning by binding to NPR1, an ANP receptor. However, the expression of ANP in adipocytes has not yet been studied. Therefore, in this study, we investigate the expression of ANP in beige-like adipocytes induced by docosahexaenoic acids (DHA), T3, or a PPAR agonist, rosiglitazone. First, we found that brown adipocyte-specific genes were upregulated in beige-like adipocytes. DHA promoted ANP expression in beige-like cells, whereas DHA-induced ANP expression was abolished by GPR120 knockout. ANP secretion of beige-like adipocytes was increased via PKC/ERK1/2 signaling in the GPR120 pathway. Furthermore, ANP secreted from beige-like adipocytes acted on HEK-293 cells, the recipient cells, leading to increased cGMP activity. After the NPR1 knockdown of HEK-293 cells, cGMP activity was not changed. Taken together, our findings indicate that beige-like adipocytes induce ANP secretion, which may contribute to improving obesity-associated metabolic disease.

## 1. Introduction

White adipose tissue, the major adipose organ in mammals, is specialized for the storage of excess energy [1]. Most mammals also have another type of adipocyte tissue that plays a role in adaptive thermogenesis. The two subtypes of thermogenic adipocytes are brown and beige fat cells [2]. Brown adipocytes are established prenatally within brown fat depots [2,3]. In contrast, beige adipocytes are induced sporadically within white adipose tissue via cold exposure as well as other types of stimuli (e.g., chemical compounds and genetic factors) [2,3]. These thermogenic functions are mediated predominantly by the presence of UCP1, a protein that catalyzes a proton leak across the inner mitochondrial membrane [4,5]. Brown and beige adipocytes prevent hypothermia and obesity by breaking fat down to produce heat [4].

Several studies have reported that omega-3 unsaturated fatty acids, such as eicosapentaenoic acid (EPA) or docosahexaenoic acid (DHA), induce thermogenic functions to activate brown adipocytes or convert white fat cells into beige fat cells [6]. It has also been found to be associated with G-protein coupled receptor 120 (GPR120), an omega-3 unsaturated fatty acid receptor [7]. GPR120 activates heterodimer Gaq subunits to induce thermogenic functions through secondary messengers such as Ca^2+^, cAMP, and diacylglycerol [8,9]. Treatment with omega-3 or the GPR120 agonist increases the thermogenic energy expenditure in high fat diet-induced obese rat models, reducing obesity and metabolic dysfunction. [10,11].

Corin, a transmembrane serine protease, is mainly distributed in the heart, kidney, and bone [12,13]. It is responsible for converting inactive pro-atrial natriuretic peptide (pro-ANP) into the active form of ANP [14]. The tissue expression patterns of corin and natriuretic peptides are very similar [15]. Activated ANP is a peptide hormone consisting of 28 amino acids that binds to NPR1, a receptor in target organs such as the kidneys and peripheral blood vessels, converting intracellular GTP into cGMP to promote the excretion of Na^+^, inhibit Na^+^ reuptake, and induce vasodilation [16,17]. Recently, ANP was found to act on adipocytes to increase mitochondrial biogenesis and UCP1 gene expression, inducing heat generation by converting adipocytes into beige fat [18,19]. Although ANP expression and secretion are known to occur mainly in the heart [20,21], it has been reported that ANP gene expression also occurs in adipocytes [22]. When ANP genes were expressed in human preadipose immortalized (Chub-S7) cells and differentiated Chub-S7 adipocytes, ANP expression was not expressed in differentiated adipocytes, although ANP expression and secretion was observed in preadipocytes. There does not currently exist any studies on ANP expression and secretion in beige adipocytes. As such, in this study, we investigate whether ANP expression and secretion occurs in beige adipocytes.

## 2. Results

### 2.1. DHA-Treated Adipocytes Upregulates the Expression of Corin and ANP

**Figure 1:**
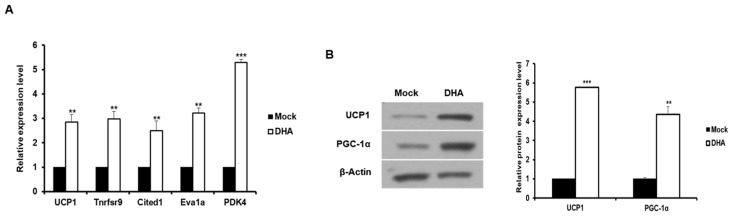
Browning of adipocytes in 3T3-L1 cells was induced by DHA. 3T3-L1 cells were exposed to DHA (100 μM) for 2 d in the presence of the differentiation medium. (**A**) The expression of brown/beige adipocytes specific genes was analyzed by qRT-PCR in the DHA-treated adipocytes. The basal delta-Ct levels for tested genes are presented as Supplementary Table S1. ** *p* < 0.01, *** *p* < 0.001. (**B**) The levels of thermogenic proteins UCP1 and PGC-1α were determined by Western blotting in 3T3-L1 adipocytes treated with DHA. ** *p* < 0.01, *** *p* < 0.001. The data are expressed as the means ± standard deviation from three or more independent experiments.

The expression of brown/beige adipocyte marker genes was first analyzed to determine whether beige adipocytes were induced by DHA treatment in 3T3-L1 cells. As expected, the mRNAs levels of brown/beige adipocyte-specific genes, namely *UCP1, Tnfrsf9, Cited1, Eva1a*, and *PDK4*, were significantly increased in the DHA-treated 3T3-L1 cells compared to the control group (Figure 1A). The protein levels of the thermogenic markers UCP1 and PGC-1α also increased when cells were treated with DHA (Figure 1B). As a result, it was confirmed that DHA induces the browning of 3T3-L1 adipocytes.

RNA-seq was performed in 3T3-L1 cells treated with DHA or the cells defected with GPR120, a receptor for omega-3 fatty acids. In RNA-seq analysis, the genes involved in cell differentiation/lipid metabolic processes were selected by gene ontology. Subsequently, 94 genes were selected resulting from an over 2-fold difference in gene expression between the DHA-treated cells and the BSA-treated cells, and a less than 0.5-fold difference in gene expression between the GPR120-deficient cells and normal cells. The genes showing a 20- or greater fold-change in DHA-treated cells compared to controls were Fam57b, Txk, Csn1s1, Skor1, Ranbp31, Plcb2, Elf5, Clec4d, Thrsp, Mboat2, and corin. Finally, corin was selected since it had the lowest read count value in white adipocytes (Figure S1).

**Figure 2:**
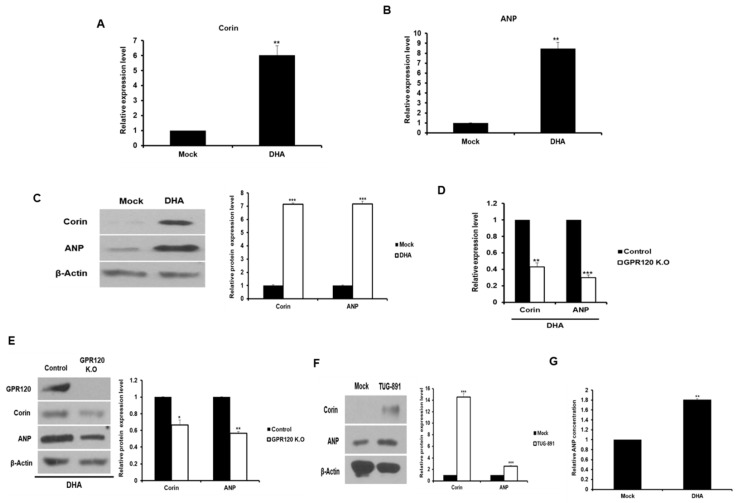
The expression of corin and ANP was increased in DHA-induced adipocytes. 3T3-L1 cells were exposed to DHA (100 μM) for 2 d in the presence of the differentiation medium. (**A**–**C**) The expression of corin and ANP in DHA-induced adipocytes was analyzed by qRT-PCR and Western blotting. The basal delta-Ct levels for tested genes are presented as Supplementary Table S2. ** *p* < 0.01. (**D**,**E**) The expression of corin and ANP was measured in the GPR120 deficient adipocytes treated with DHA by qRT-PCR and Western blotting. * *p* < 0.05, ** *p* < 0.01, *** *p* < 0.001. (**F**) 3T3-L1 cells were treated with 1 μM TUG-891, a potent GPR120 agonist for 24 h. The corin and ANP expression levels were analyzed by Western blotting. (**G**) The concentration of ANP was measured in the media derived from the DHA-induced adipocytes using ELISA. ** *p* < 0.01. The data are shown as the means ± standard deviations from three or more independent experiments.

Corin and corin-activated ANP expression were investigated in the DHA-treated 3T3-L1 adipocytes and the GPR120-deficient cells to verify the expression of *corin* selected by RNA-seq. The mRNA levels of *corin* and *ANP* in the DHA-treated adipocytes were found to be significantly increased compared to the control cells (Figure 2A,B). The expression of corin and ANP protein was also found to increase in the DHA-treated cells. (Figure 2C). The treatment of GPR120 knock out adipocytes with DHA resulted in decreased mRNA and protein levels for corin and ANP compared to the control cells (Figure 2D,E). In addition, when TUG-891, a GPR120 agonist, was treated with 3T3-L1 adipocytes, the protein levels of corin and ANP were higher than those of the control group (Figure 2F). The expression of proprotein convertase subtilisin/hexin type 6 (PCSK6), a corin activator, was also investigated. PCSK6 is known to increase mature ANP secretion [23]. As shown in Figure S2, PCSK6 expression was increased in the adipocytes treated with DHA than in the control cells. We determined the amount of ANP secreted by the DHA-treated cells. As a result, a 2-fold increase in ANP secretion was found in the cell line treated with DHA compared to the control group (Figure 2G). These results indicate that the expression and secretion of ANP increase via the GPR120 pathway in DHA-treated cells.

### 2.2. The Effect of DHA on ANP Secretion is Mediated by PKC/ERK Pathway

**Figure 3:**
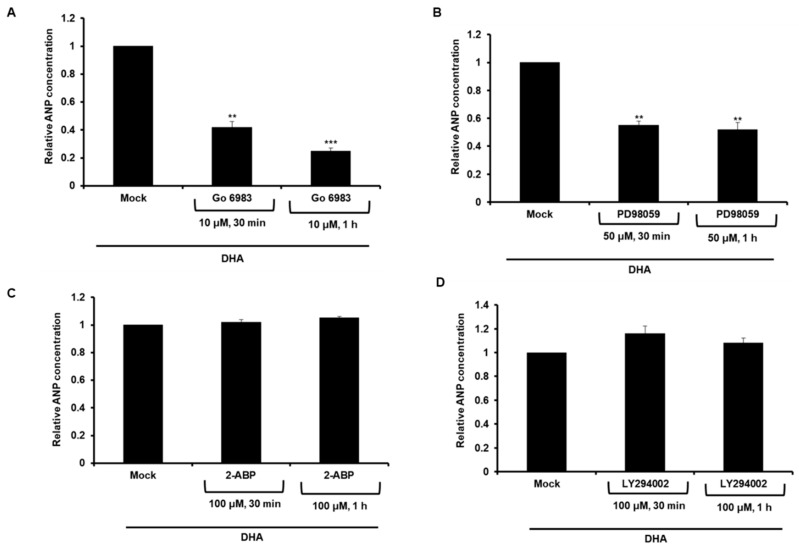
ANP secretion was mediated by the PKC/ERK 1/2 signaling pathway in DHA-induced adipocytes. 3T3-L1 cells were exposed to DHA (100 μM) for 2 d in the presence of the differentiation medium. (**A**–**D**) 3T3-L1 adipocytes were treated with DHA in the presence of (**A**) PKC inhibitor Go 6983, (**B**) ERK inhibitor PD98059, (**C**) Ca^2+^ signaling inhibitor 2-ABP, or (**D**) PI3K inhibitor LY294002. The concentration of ANP in the media of adipocytes was measured by ELISA. ** *p* < 0.01, *** *p* < 0.001. Bars are means ± standard deviations of three independent experiments.

Since ANP secretion was increased in the DHA-treated adipocytes via GPR120, we next examined which of the pathways mediated by GPR120 regulates ANP expression. The DHA-treated 3T3-L1 cells were exposed to the PKC inhibitor Go 6983, the EKR inhibitor PD98059, the Ca^2+^ signaling inhibitor 2-aminoethoxydiphenyl borate (2-ABP), or the PI3K inhibitor LY294002, respectively. The resulting levels of secreted ANP were measured. As a result, ANP secretion was found to decrease in cells treated with Go 6983, and PD98059 (Figure 3A,B); however, no differences in the cells treated with 2-ABP and LY294002 were found (Figure 3C,D). These results suggest that increased ANP secretion in the DHA-treated cells occurs via the GPR120-mediated PKC/ERK 1/2 pathway.

### 2.3. The Expression of Corin and ANP is Increased in Beige-Like Adipocytes

**Figure 4:**
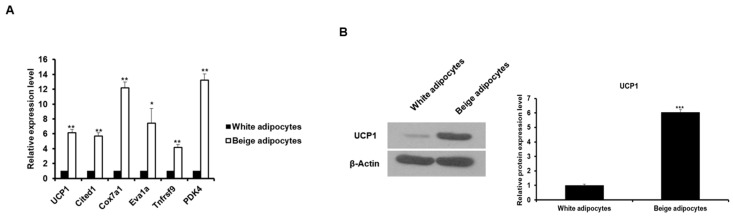
3T3-L1 cells differentiated into beige-like adipocytes in the presence of T3 and rosiglitazone. (**A**) The expression of brown/beige adipocytes-specific genes was determined by qRT-PCR in white adipocytes (mature 3T3-L1 adipocytes) and beige adipocytes (3T3-L1 adipocytes exposed to T3 and rosiglitazone). The basal delta-Ct levels for tested genes are presented as Supplementary Table S3. * *p* < 0.05, ** *p* < 0.01. (**B**) The expression of UCP1 in white adipocytes and beige adipocytes was analyzed by Western blotting. The level of UCP1 protein was used as a marker for beige adipocytes. *** *p* < 0.001. The data are shown as the means ± standard deviations of three independent experiments.

**Figure 5:**
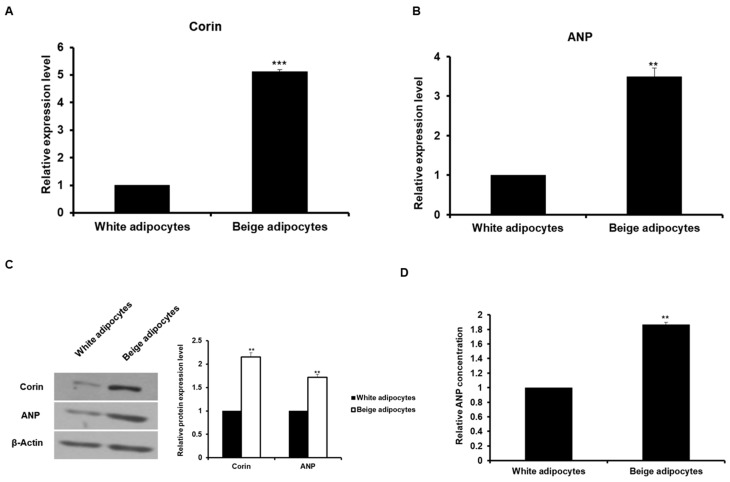
The expression of corin and ANP was increased in beige-like adipocytes induced by T3 and rosiglitazone. (**A**–**C**) The expression of corin and ANP in white adipocytes and beige adipocytes was analyzed by qRT-PCR and Western blotting. The basal delta-Ct levels for tested genes are presented as Supplementary Table S4. ** *p* < 0.01, *** *p* < 0.001. (**D**) The concentration of ANP was measured in the media of adipocytes by ELISA. ** *p* < 0.01. The representative data are presented as the mean ± standard deviation of three independent experiments.

Next, we examined whether both corin and ANP are expressed in beige-like adipocytes induced by other conditions except DHA. Referring to the results of a previous study [24], we found that beige adipocytes are induced by treatment with tridothyronine (T3) and a PPAR agonist, rosiglitazone. As shown in Figure 4A, the mRNA expression of brown/beige fat cell marker genes was increased in 3T3-L1 cells treated with T3 and rosiglitazone compared to white adipocytes. In addition, UCP1 protein expression was higher in beige-like adipocytes than in white adipocytes (Figure 4B). The *corin* and *ANP* mRNA levels were analyzed in T3 and rosiglitazone-induced beige adipocytes. As a result, the expression of these genes was significantly increased in beige-like adipocytes compared to white adipocytes (Figure 5A,B). The protein levels of these genes were also found to increase in beige-like adipocytes (Figure 5C). The secretion of ANP in beige-like adipocytes was about 2-fold higher than in white adipocytes (Figure 5D). Interestingly, the expression of an ANP receptor *NPR1* was also increased in beige-like adipocytes compared to white adipocytes (Figure S2). These results indicate that corin and ANP expression is increased in beige-like adipocytes induced by conditions other than treatment with DHA.

### 2.4. ANP Derived from Beige-Like Adipocytes Enhances cGMP Levels in HEK-293 Cells

**Figure 6:**
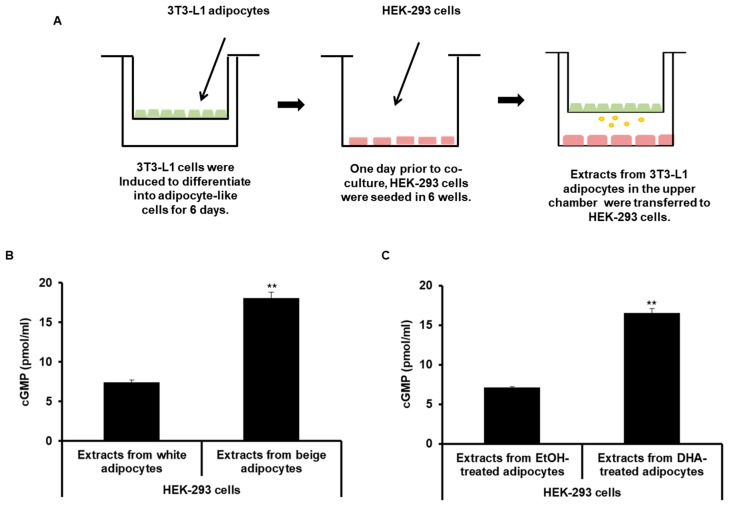
ANP secreted from beige-like adipocytes increased the levels of cGMP in HEK-293 cells. (**A**) Schematic representation of the in vitro co-culture system. 3T3-L1 white adipocytes, beige adipocytes, or DHA-treated adipocytes were grown in 6-well culture inserts with a semipermeable support membrane. HEK-293 cells were cultured in the 6-well culture plates. Culture inserts containing 3T3-L1 adipocytes were placed in the 6-well plates to establish the co-culture system for 24 h. (**B**) HEK-293 cells were co-cultured with 3T3-L1 white adipocytes or beige adipocytes exposed to T3 and rosiglitazone. The cGMP concentrations were analyzed in HEK-293 cells. (**C**) HEK-293 cells were co-cultured with DHA-treated adipocytes, and the cGMP levels in HEK-293 cells were measured. ** *p* < 0.01. Bars are means ± standard deviations of three independent experiments.

**Figure 7:**
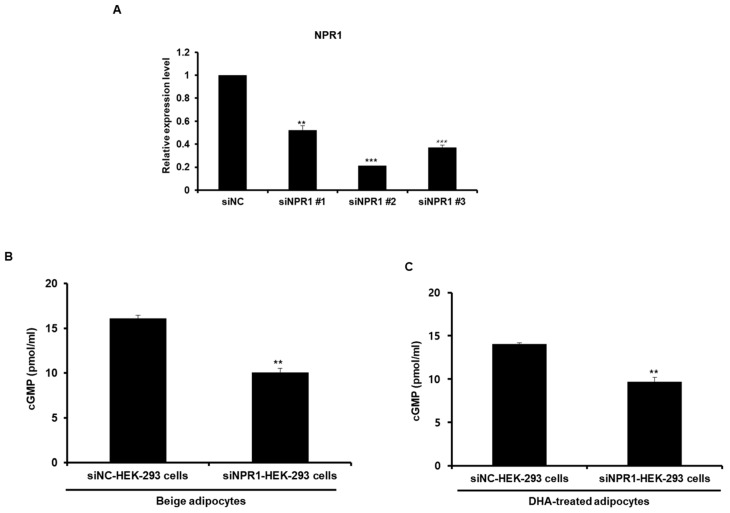
ANP secreted from beige-like adipocytes induced an increased in the concentration of cGMP in HEK-293 cells via the ANP receptor NPR1. (**A**) After transfection with three different NPR1 siRNA oligos, the cells were analyzed for the levels of NPR1 mRNA. ** *p* < 0.01, *** *p* < 0.001. (**B**,**C**) HEK-293 cells were transfected with siNPR1 oligos or the siRNA negative control, then co-cultured with beige-like adipocytes induced by T3/rosiglitazone or DHA. The cGMP levels in the HEK-293 cells were determined by cGMP immunoassay. ** *p* < 0.01. Bars are means ± standard deviations of three independent experiments.

We investigated whether ANP secreted from beige-like adipocytes acted on recipient cells. Renal cells are known to have an ANP receptor NPR1. HEK-293 cells were cultured in 6-well plates, then transwell inserts with beige-like adipocytes were placed on them for 24 h, as shown in Figure 6A. Beige-like adipocytes were induced by treatment with T3 and rosiglitazone or DHA. After co-culturing, HEK-293 cells were collected and examined for the levels of cGMP as a secondary messenger. As a result, the levels of cGMP were found to be increased in HEK-293 cells co-cultured with beige-like adipocytes compared to HEK-293 cells co-cultured with white adipocytes (Figure 6B). In addition, the concentration of cGMP was increased in HEK293 cells co-cultured with the DHA-treated adipocytes compared to HEK293 cells co-cultured with the BSA-treated adipocytes (Figure 6C). Next, co-cultures with both beige-like adipocytes and HEK-293 cells expressing knocked-down NPR1, an ANP receptor, were performed to determine whether the increased concentration of cGMP in HEK-293 cells resulted directly from the ANP secreted by the adipocytes. Firstly, several NPR1 siRNAs were constructed to investigate which one was the most efficient siRNA oligo for decreasing NPR1 expression in HEK-293 cells. siNPR1 # 2 was found to significantly decrease the expression of the NPR1 gene and was used for the subsequent experiment (Figure 7A). NPR1 knocked-down HEK-293 cells were co-cultured with adipocytes treated with T3 and rosiglitazone, resulting in decreased cGMP concentrations compared to the controls (Figure 7B). NPR1 knocked-down HEK-293 cells incubated with DHA-treated adipocytes also showed lower levels of cGMP than the controls (Figure 7C).

Taken together, our results suggest that beige-like adipocytes express and secrete ANP. The secreted ANP appears to act on the recipient cells.

## 3. Discussion

White adipose tissue is an endocrine organ that releases hormones called adipokines. Brown adipose tissue also secretes hormones called batokines. Several researchers have studied the hormones released when adipose tissues are browned [11,25,26,27,28]. For example, fibroblast growth factor 21 (FGF21) is a secreted factor induced by noradrenergic cAMP-mediated mechanisms in brown adipocytes [26]. FGF21 has important effects on energy balance, glucose metabolism, and lipid metabolism. FGF21 increases the expression of PGC-1α and UCP1 in adipocytes and promotes thermogenesis [11]. In addition, growth and differentiation factor 15 (GDF15) is secreted from brown and beige adipocytes that undergo noradrenergic, cAMP-mediated thermogenic activation [27]. This targets macrophages and downregulates the expression of proinflammatory genes. The treatment of FGF21 with brown adipocytes increases GDF15 secretion [27]. In humans, interleukin-6 (IL-6) is secreted from beige adipocytes more than white adipocytes [28]. IL-6 secreted from beige adipocytes acts as an autocrine to induce the browning of white adipocytes [28]. When browning from white fat, batokines secreted as an autocrine act on adipocytes to induce beige adipocytes or other target cells. In this study, we observed that ANP was secreted in adipocytes with brown/beige phenotypes rather than white fat cells. The secreted ANP as a paracrine agent acted on the kidney cells and increased the cGMP levels. Many batokines act on adipocytes as autocrine [11,28]. In this study, we found that NPR1 expression was higher in beige adipocytes than in white adipocytes. Therefore, ANP secreted from adipocyte may act as autocrine. ANP activity depends on the ratio of ANP receptor NPR1 and clearance receptor NPR3 [29]. Obesity-induced rats fed a high-fat diet have a lower NPR1/NPR3 ratio than the normal diet [30]. In addition, mice knocked out of NPR3 have similar effects to ANP-induced brown fat thermogenic program [31]. In order to improve obesity, the NPR1/NPR3 ratio must be high in adipose tissue. Therefore, ANP secreted from beige adipocytes with high NPR1 expression can act as an autocrine and induce thermogenesis. These results indicate that various batokines secreted from brown/beige fat are involved in maintaining the homeostasis in the body.

Omega-3 fatty acids have been known to promote the activation of brown and beige fats, having been used as novel treatments for obesity and other metabolic diseases [6]. In this study, 3T3-L1 adipocytes were treated with DHA to increase the expression of beige adipocyte-specific genes. In addition, this study also observed that ANP expression was increased by DHA. We investigated whether increased ANP expression in DHA-induced beige fat was observed in cells derived from beige fat under different conditions, such as treatment with T3 and rosiglitazone. As a result, it was observed that ANP expression was increased in the beige adipocytes induced by T3 and rosiglitazone. Since rosiglitazone is a PPARγ agonist and PPARγ is expressed highly in beige adipocytes [32,33,34], we investigated whether ANP expression is directly regulated by PPARγ. ChIP assays were performed due to the potential PPARγ binding sites existing on the ANP promoter. However, PPARγ was not bound to the ANP promoter (data not shown). This confirmed that PPARγ is not directly involved in the transcriptional regulation of ANP expression in beige adipocytes, suggesting that ANP expression may be regulated by other intracellular regulatory mechanisms.

Although there are currently no studies on the association between omega-3 fatty acids andANP in beige adipocytes, the effect of DHA-induced ANP expression on cardiac fibroblasts has been previously investigated, wherein no changes in ANP mRNA in cardiac fibroblasts treated with EPA (10 μM) or DHA (10 μM) were found [35]. In rats, the expression of ANP in the cardiac tissues did not vary when fed with DHA and EPA compared to a standard chow diet [36], suggesting that omega-3 fatty acids do not induce ANP expression in cardiac fibroblasts. In this study, however, the DHA treatment of adipocytes increased the expression of ANP via the GPR120-mediated pathway. GPR120 expression is known to be high in beige adipocytes [11], but low in the heart. [37]. Therefore, in rats fed DHA or EPA, there is no difference in ANP expression due to the low expression of GPR120 in the heart. These results indicate that DHA regulates ANP expression in a cell-specific manner.

GPR120, an omega3 fatty acid receptor, stimulates beige adipocytes and increases FGF21 expression and secretion [11]. Mice with knocked-out GPR120 are found to reduced fat with browning phenotype and decreased UCP1 expression in their adipose tissue. In the fat cells of mice with knocked-out GPR120, the expression and secretion of FGF21 mRNA are reduced after treatment with omega-3 fatty acids compared to wild-type mice [11]. FGF21 expression induced by GPR120 in adipocytes is known to occur via the ERK1/2 pathway [7]. In addition to FGF21, batokines induced by GPR120 have not been well characterized. In this study, we found that ANP synthesis and secretion were regulated by GPR120. It was also observed that the ANP pathway is secreted by GPR120 via the ERK1/2 pathway. Therefore, in addition to FGF21, ANP was elucidated as another batokine mediated by GPR120.

Overall, the expression and secretion of ANP increased during the browning of white adipose cells. These findings provide a basis for the development of therapeutic strategies for the treatment of metabolic diseases, including obesity.

## 4. Materials and Methods

### 4.1. Cell Culture and Adipocyte Differentiation

3T3-L1 cells and HEK293 cells were provided by ATCC. 3T3-L1 cells were cultured in Dulbecco’s modified Eagle’s medium (DMEM, Hyclone, Logan, UT, USA) containing 10% bovine calf serum (Hyclone, Logan, UT, USA) at 37 ℃ in a 5% CO_2_ incubator. HEK293 cells were grown in Eagle’s minimum essential medium (MEM) supplemented with 10% fetal bovine serum (FBS; Hyclone, Logan, UT, USA). 3T3-L1 cells were cultured on 6-well plates at a density of 3 × 10^5^ cells per well. To induce adipocyte differentiation, 3T3-L1 preadipocytes were maintained until confluence. After two days, the medium was changed to differentiation medium using isobutylmethylxanthine (IBMX), insulin, dexamethasone (Sigma-Aldrich, St. Louis, MO, USA) or DHA (Cayman Chemical, Ann Arbor, MI) for 2 d, followed by the differentiation medium supplemented 10% FBS, insulin or DHA for 2 d. The medium was renewed every other day until day 6. To induce the browning of 3T3-L1 adipocytes, differentiation induction medium was supplemented with tridothyronine (T3) and rosiglitazone (Sigma-Aldrich, St. Louis, MO, USA).

For the co-culture experiments, 0.4 μm pore size transwell chambers (Corning Inc., Corning, NY, USA) were used to avoid direct contact between 3T3-L1 adipocytes and HEK-293 cells. 3T3-L1 cells were added in the upper chamber of a 6-well plate and were differentiated with DHA or differentiation reagents. Two days before the cGMP assay, HEK-293 cells were seeded in a 6-well plate at a density of 2 × 10^5^ cells per well, rather than in plates with 3T3-L1 cells. The differentiated 3T3-L1 cells in the upper chamber were transferred to a plate with the HEK-293 cells. After 3T3-L1 and HEK-293 cells were co-cultured for 24 h, the HEK-293 cells were harvested. HEK-293 cells were analyzed cGMP activity using the parameter cGMP immunoassay (R&D systems, Minneapolis, MN, USA) according to the protocol provided by the manufacturer.

To obtain GPR120 knockout adipocytes, 3T3-L1 cells were transfected with pCMV-Cas9-GFP plasmids containing gRNA specific for GPR120 genes (Sigma-Aldrich, St. Louis, MO, USA) using lipofectamine 2000 (Invitrogen, Carlsbad, CA, USA). GFP-expressing cells were sorted using the BD Aria flow cytometer (BD Bioscience, San Jose, CA). After sorting, 3T3-L1 cells were cultured, and adipose differentiation was induced. GPR120 expression was examined by Western blotting.

### 4.2. RNA-Seq Analysis

The samples used for the RNA-seq analysis were the 3T3-L1 cells treated with DHA and GPR120 knockout adipocytes. As a control group, the 3T3-L1 cells treated with bovine serum albumin (BSA, Sigma-aldrich, St. Louis, MO, USA) and differentiated adipocytes were used. Total RNA was extracted and submitted to eBiogen Inc (Seoul, Republic of Korea). Library preparation and RNA sequencing were performed using the RNA-seq services provided by Ebiogen Inc (Seoul, Republic of Korea). Briefly, library construction was conducted using a SMARTer Stranded RNA-seq kit (Illumina, San Diego, CA, USA) according to the manufacturer’s protocol. Sequencing was performed by paired-end sequencing using HiSeq 2500 (Illumina, San Diego, CA, USA). Functional category analysis was carried out by determining the Gene Ontology (GO) terms that were differentially expressed genes (DEGs). Two gene categories that have direct or indirect effects on adipocyte differentiation and adipocyte metabolism were selected. The categories were as follows: (1) cell differentiation and (2) lipid metabolic process. Every gene expression value was measured. Among them, those with an over 2-fold change in the DHA group compared to the control (BSA) group and under half-fold change in the GPR120 knockout adipocytes group compared to control (adipocytes) group were selected.

### 4.3. Quantitative Real-Time Reverse Transcription Polymerase Chain Reaction (qRT-PCR)

The total RNA was isolated from 3T3-L1 cells using the TRIzol reagent (Invitrogen, Carlsbad, CA, USA). The RNA was converted into cDNA using MMLV-reverse transcriptase (#M1705, Promega, Madison, WI, USA) and random primers (Invitrogen, Carlsbad, CA, USA) according to the manufacturer’s instructions. Real-time quantitation was performed using a Roto gene Q PCR instrument (Qiagen, Hilden, Germany). PCR was performed under the following conditions: 10 min at 95 °C followed by 40 cycles of 15 s at 95 °C, 10 s at 60 °C, 15 s at 72 °C, and 5 min at 72 °C. The reverse transcription reaction mixture was used with the SYBR Green PCR Master mix (Bioline, Seoul, Republic of Korea). PCR reactions were run in triplicate for each sample. The relative expression level of each gene was analyzed using the 2^−ΔΔCt^ method. Actin was used for the normalization of gene expression. The sequences of all primers used in qRT-PCR are provided in Table 1.

### 4.4. Western Blot Analysis

Cells were lysed with lysis buffer (50 mM Tris-HCl, 150 mM NaCl, 1% Nonidet P-40, 0.1% SDS, protease inhibitor cocktail, 50 mM NaF, and 0.2M Na_3_VO_4_). The protein concentration in the supernatants was measured using the Bradford assay (Bio-Rad, Hercules, CA, USA). Proteins extracts were separated on a 12% SDS polyacrylamide gel and transferred onto a nitrocellulose membrane. After blocking with 5% skimmed milk in phosphate-buffered saline with Tween-20 (PBST) for 1 h at room temperature, the membranes were probed with anti-UCP1 (#ab155117, Abcam, Cambridge, MA, USA), anti-ANP (sc515701, Santa Cruz Biotechnology, Santa Cruz, CA, USA), anti- PGC1α (#M00236, Bosterbio, Pleasanton, CA, USA), anti-Corin (#PA5-42916, Novus, Littleton, CO, USA), GPR120 (#NBP1-00858, Novus, Littleton, CO, USA) and anti-actin (#A2060, Sigma-Aldrich, St. Louis, MO, USA). After washing, the protein extracts were incubated with horseradish peroxidase-conjugated secondary antibodies and detected using an enhanced chemiluminescence substrate (Amersham Biosciences, Freiburg, Germany).

### 4.5. Enzyme-Linked Immunosorbent Assay (ELISA)

The concentration of ANP in 3T3-L1 adipocytes was quantified using the ANP ELISA kit (Abnova, Taipei City, Taiwan) according to the manufacturer’s instructions. Briefly, 3T3-L1 adipocytes were incubated in serum-free medium overnight. Cell media was added to the wells coated with anti-ANP antibody. Then, a horse peroxidase colorimetric reaction was performed. The immunoassay directed against the C-terminal region of ANP detects both pro-ANP and ANP. Finally, the absorbance was measured at 450 nm using a Vmax microplate spectrophotometer (Molecular Devices, Sunnyvale, CA, USA).

### 4.6. Statistical analysis

Data are expressed as the mean ± standard error of the mean (SEM). Differences between groups were analyzed using Student’s *t*-test. Values of *p* < 0.05 and *p* < 0.01 were considered as statistically significant. Statistical analyses were performed using SPSS version 11.0 (SPSS, Chicago, IL, USA).

## Figures and Tables

**Table 1:** Primer sequences used for qRT-PCR.

Gene	Sequence
*β-actin*	(F)5′-GTGACGTTGACATCCGTAAAGA-3′
	(R)5′-GCCGGACTCATCGTACTCC-3′
*UCP1*	(F)5′-AGGCTTCCAGTACCATTAGGT-3′
	(R)5′-CTGAGTGAGGCAAAGCTGATTT-3′
*Tnfrsf9*	(F)5′-CGTGCAGAACTCCTGTGATAA C-3′
	(R)5′-GTCCACCTATGCTGGAGAAGG-3′
*Cited1*	(F)5′-AACCTTGGAGTGAAGGATCGC-3′
	(R)5′-GTAGGAGAGCCTATTGGAGATGT-3′
*Eva1a*	(F)5′-GGGGAGACCGAAGGAAATGAGA-3′
	(R)5′-CTCCAGCCCTGCACACTCTA-3′
*PDK4*	(F)5′-AGGGAGGTCGAGCTGTTCTC-3′
	(R)5′-GGAGTGTTCACTAAGCGGTCA-3′
*Cox7a1*	(F)5′-GCTCTGGTCCGGTCTTTTAGC-3′
	(R)5′-GTACTGGGAGGTCATTGTCGG-3′
*Corin*	(F)5′-GCTGGTGACTGCTAACTTGCT-3′
	(R)5′-CCCATCAGTGACCAAAGGTTC-3′
*ANP*	(F)5′-TCGTCTTGGCCTTTTGGCT-3′
	(R)5′-TCCAGGTGGTCTAGCAGGTTCT-3′
*NPR1*	(F)5′-GGGATACAGTCAACACAGCCTCAA-3′
	(R)5′-CGAAGCTCCAGCTCGAAA C-3′

## Supplementary Materials

Supplementary materials can be found at https://www.mdpi.com/1422-0067/20/24/6128/s1.
