# Supplementary material for: Expression and Secretion of an Atrial Natriuretic Peptide in Beige-Like 3T3-L1 Adipocytes
Source: Int J Mol Sci. 2019 Dec 5;20(24):6128. doi: 10.3390/ijms20246128 (PMC6940835; doi:10.3390/ijms20246128)
Supplement: Supplementary file 1 [file ijms-20-06128-s001.pdf]

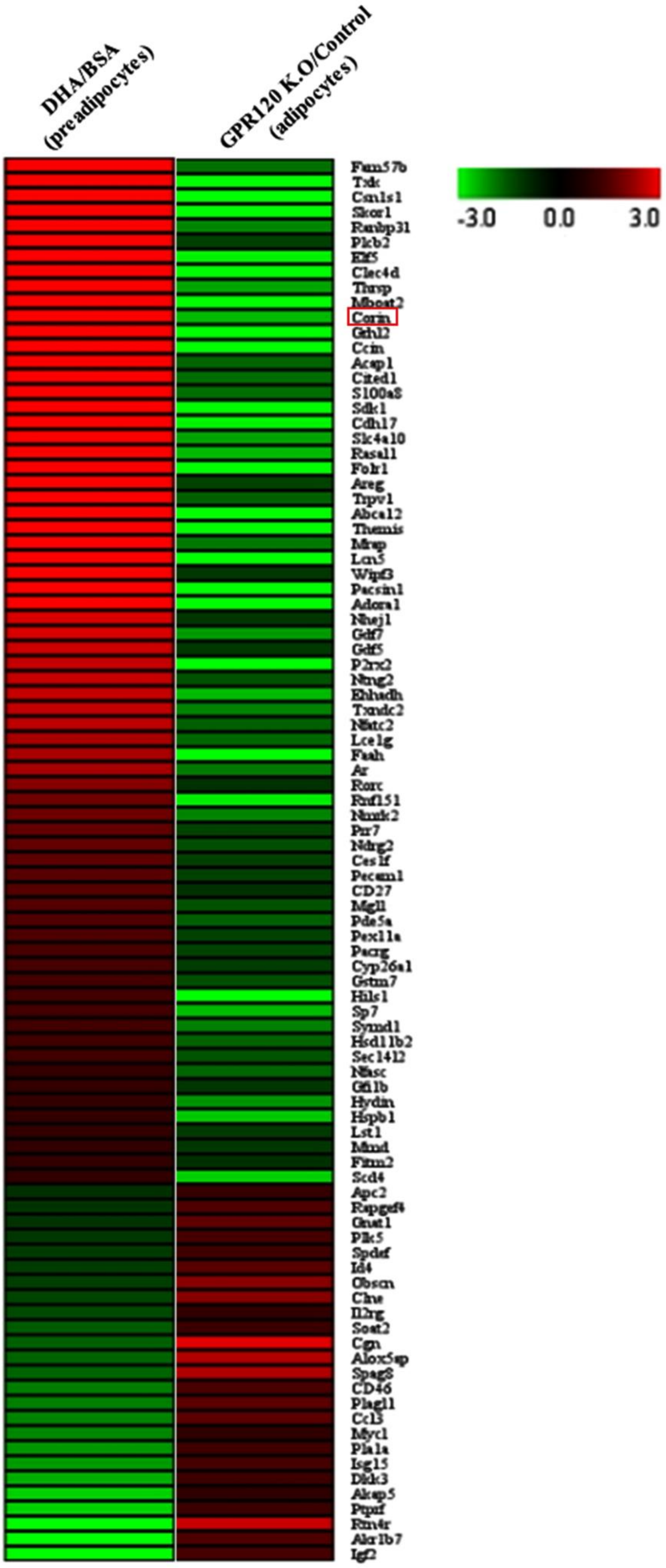

Supplementary Figure 1. Comparison of gene expression in DHA-treated or GPR120-deficient cells. 3T3-L1 preadipocytes were treated with 100 μM of DHA for 24 h. In RNA-seq analysis, the genes

belonging to cell differentiation/lipid metabolic process were selected from gene ontology. Subsequently, 94 genes were selected resulting from an over two-fold difference in gene expression between the DHA-treated cells and the BSA-treated cells, and a less than 0.5-fold difference in gene expression between the GPR120-deficient cells and normal cells.

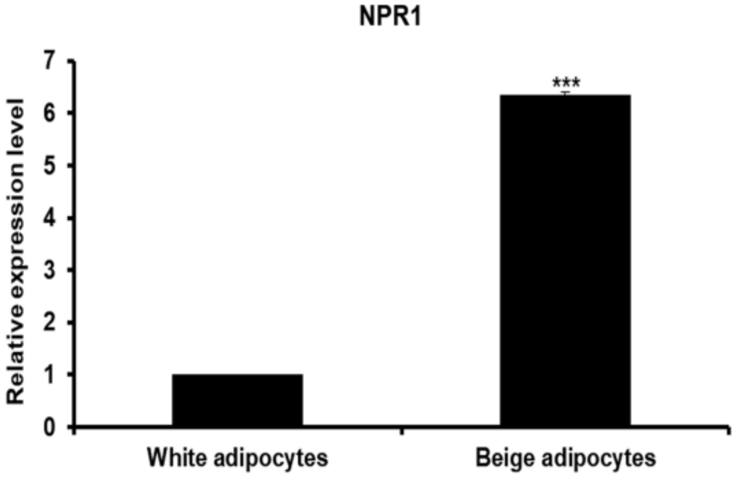

Supplementary Figure 2. The expression of NPR1 was increased in beige-like adipocytes induced by T3 and rosiglitazone. The expression of NPR1 mRNA in white adipocytes and beige adipocytes was analyzed by qRT-PCR.*** $p < 0.001$.

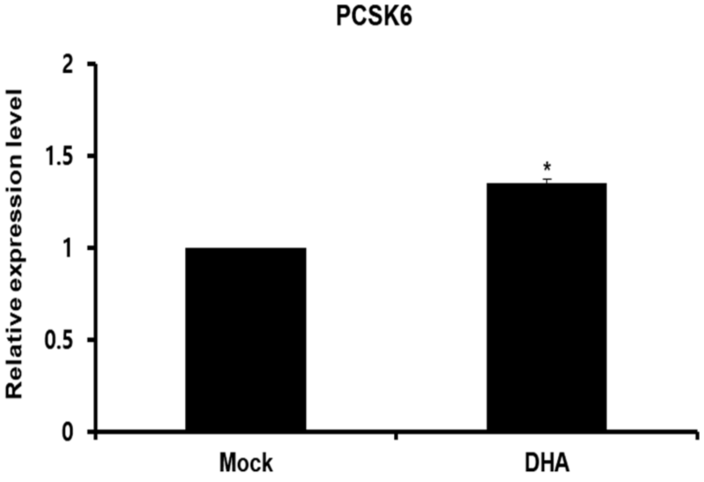

Supplementary Figure 3. The expression of PCSK6 was increased in DHA-induced adipocytes. 3T3-L1 cells were exposed to DHA (100 μM) for 2 d in the presence of the differentiation medium. The expression of PCSK6 in DHA-induced adipocytes was analyzed by qRT-PCR. * $p < 0.05$.

Supplementary Table 1. Ct values of each gene present in Figure 1.

Gene	Average Ct value		Delta Ct value	
	**Mock**	**DHA**	**Mock**	**DHA**
Actin	18.53	18.54	-	-
UCP1	30.33	28.82	11.8	10.28
Tnfrsf9	27.07	25.52	8.54	6.98
Cited1	27.63	26.34	9.1	7.8
Eva1a	27.58	25.88	9.05	7.34
PDK4	26.21	23.81	7.68	5.27

Supplementary Table 2. Ct values of each gene present in Figure 2

**Figure 2A & B**				
**Gene**	**Average Ct value**		Delta Ct value	
	**Mock**	DHA	Mock	DHA
Actin	17.51	17.35	-	-
Corin	31.99	29.17	14.48	11.82
ANP	31.48	28.24	13.97	10.89
Figure 2D				
Gene	Average Ct value		Delta Ct value	
	Control	GPR120 K.O	Control	GPR120 K.O
Actin	17.25	17.40	-	-
Corin	29.16	30.56	11.91	13.16
ANP	28.32	30.22	11.07	12.82

Supplementary Table 3. Ct values of each gene present in Figure 4

**Gene**	**Average Ct value**		**Delta Ct value**	
	**White adipocytes**	**Beige adipocytes**	**White adipocytes**	**Beige adipocytes**
Actin	17.25	17.15	-	-
UCP1	30.53	27.77	13.28	10.62
Cited1	28.32	25.75	11.07	8.6
Cox7a1	28.32	24.58	11.07	7.43
Eva1a	28.65	25.66	11.4	8.51
Tnfrsf9	27.22	25.07	9.97	7.92
PDK4	26.25	22.41	9	5.26

Supplementary Table 4. Ct values of each gene present in Figure 5

**Figure 5A & B**				
**Gene**	**Average Ct value**		Delta Ct value	
	**Mock**	DHA	Mock	DHA
Actin	17.94	18.01	-	-
Corin	31.29	28.9	13.35	10.89
ANP	29.53	27.93	11.59	9.92
